# Systematic Study of Resveratrol Nanoliposomes Transdermal Delivery System for Enhancing Anti-Aging and Skin-Brightening Efficacy

**DOI:** 10.3390/molecules28062738

**Published:** 2023-03-17

**Authors:** Xinchao Zhang, Siyuan Chen, Dan Luo, Dan Chen, Hong Zhou, Shuting Zhang, Xuan Chen, Wangwang Lu, Wei Liu

**Affiliations:** 1 College of Life Science and Technology, Huazhong University of Science and Technology, Wuhan 430074, China; 2 College of Materials Science and Engineering, Suqian Advanced Materials Industry Technology Innovation Center, Nanjing Tech University, Nanjing 211816, China; 3 National Engineering Research Center for Nanomedicine, Huazhong University of Science and Technology, Wuhan 430075, China; 4 Guangzhou Jiyan Cosmetics Technology Co., Ltd., Guangzhou 510275, China

**Keywords:** resveratrol nanoliposomes, transdermal delivery, 3D skin model, anti-aging, skin-brightening

## Abstract

Due to the stratum corneum barrier, resveratrol is difficult to be absorbed transdermally, limiting its anti-aging and skin-brightening effects. Furthermore, there is a lack of systematic studies on the efficacy of resveratrol in human skin, especially in three-dimensional skin models and clinical trials. To overcome the low transdermal delivery issue, we encapsulated resveratrol into nanoliposomes using the high-pressure homogenization method to develop an efficient transdermal drug delivery system, and systematically evaluated its anti-aging and skin-brightening efficacy via cell line models, a three-dimensional skin model and human skin. The resveratrol nanoliposomes effectively improved the transdermal penetration and retention of resveratrol and enhanced cellular uptake. In addition, compared to free resveratrol, resveratrol nanoliposomes remarkably enhanced the skin-care effects by promoting the antioxidant capacity and collagen synthesis, inhibiting the secretion of matrix metalloproteinases, tyrosine activity and melanin synthesis. Notably, human clinical trials proved the anti-wrinkle and skin-brightening effectiveness of resveratrol nanoliposomes. Three levels of systematic studies indicated that resveratrol nanoliposomes could be a promising transdermal drug delivery system to enhance the anti-aging and skin-brightening effects of resveratrol.

## 1. Introduction

Resveratrol (Res), a kind of polyphenols stilbenoids compound, has been detected in more than 70 plant species [1]. Res exhibited various biological effects, such as antioxidant, anti-inflammatory, antibacterial, etc., making it an attractive ingredient for human skin care, especially in the fields of anti-aging and skin-brightening [2,3,4]. Specifically, Res can regulate the expression of related receptors to delay aging and inhibit tyrosinase activity to suppress melanin deposition [5,6,7]. However, its transdermal application was greatly hampered by its low solubility and poor skin penetration and retention caused by the stratum corneum barrier [8,9]. This makes it difficult for Res to reach skin tissue and be absorbed by skin target cells, severely limiting its bioavailability and transdermal application.

Regarding this limitation, one strategy is to encapsulate Res into nanocarriers to improve its solubility and stability, reduce its toxicity, enhance percutaneous permeability and regulate its drug-release behaviors [10,11,12,13]. Among various nanocarriers, nanoliposomes (NLPs) display numerous advantages and have been successfully transformed industrially [14,15,16]. NLPs are formed by FDA-approved cholesterol and phospholipids, resulting in excellent biocompatibility and low toxicity. Moreover, the unique hydrophilic core and lipid bilayer nanostructure of NLPs improves drug solubility and bioavailability by efficient loading of hydrophilic drugs into the core cavity and encapsulating hydrophobic drugs into the phospholipid bilayers [17,18,19]. In addition, the nano size and lipid bilayer structure of nanoliposomes can enhance their ability to penetrate the skin stratum corneum to promote transdermal penetration [20,21]. In our group, we have successfully developed several NLPs-based delivery systems with enhanced transdermal delivery efficiency. For example, we co-loaded copper peptide, acetyl tetrapeptide-3 and myridamoyl five-peptide-4 into NLPs to improve the stability of active peptides, achieve the multi-target synergistic effect of multiple bioactive peptide combinations, enhance the transdermal delivery efficiency and promote hair growth [18]. In addition, we developed NLPs loaded with a variety of bioactive peptides, demonstrating that NLPs can significantly improve drug uptake by relevant target cells [22]. The cationic phenethyl resorcinol encapsulated NLPs developed by us displayed a slow-release profile with significantly improved skin retention as well as remarkably boosted transdermal permeability [23]. These remarkable properties of NLPs suggest their suitability for transdermal delivery of Res.

The anti-aging and skin-brightening effects of Res as an antioxidant and tyrosinase inhibitor have been reported [24,25]. However, these studies were mainly limited to the cellular level, and systematic studies on the anti-aging and skin-brightening of Res, especially in 3D skin models and clinical trials are limited [24]. Due to the complex structure and high internal coordination of the skin, a single-cell model cannot fully reflect the drug interaction with the skin [26,27]. It is helpful and necessary to carry out efficacy studies in more complex models to clarify the mechanism of the anti-aging and skin-brightening action of Res. Based on the previous works of our group, herein, we reported Res-loaded NLPs (Res-NLPs) with high transdermal delivery efficiency, and further systematically investigated their skin-care efficacy at three different levels: cellular level, 3D skin model and human skin.

## 2. Results and Discussion

### 2.1. Characterization

**Figure 1:**
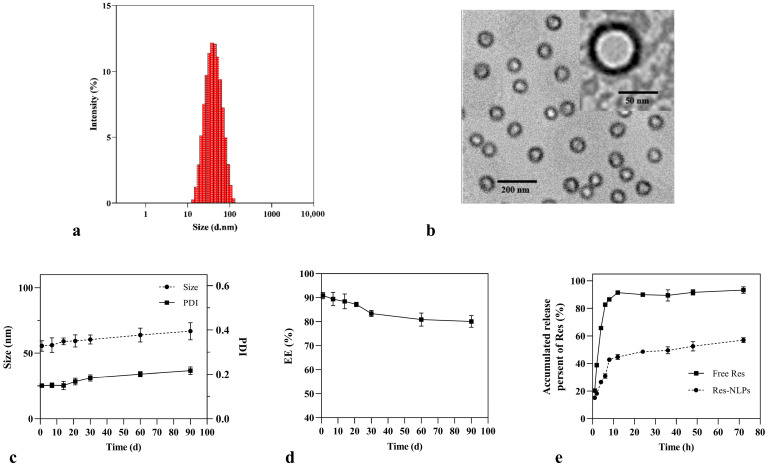
(**a**) The particle size distribution of the Res-NLPs. (**b**) TEM image of the Res-NLPs, scale bar: 200 and 50 nm. (**c**) The particle size and PDI stability of the Res-NLPs stored at 4 °C for 90 days. (**d**) The EE stability of the Res-NLPs stored at 4 °C for 90 days. (**e**) In vitro release of Res from the free Res and the Res-NLPs. Mean ± SD (*n* = 3).

The particle size and stability affect the transdermal permeation behavior of NLPs, and therefore a series of characterization studies were conducted [12]. The average particle size, polydispersity index (PDI) and zeta potential of the prepared Res-NLPs were 55.6 ± 3.2 nm, 0.149 ± 0.005 and −20.3 ± 0.8 mV, respectively (Figure 1a). Less than 100 nm particle size is a suitable size for skin surface adhesion and stratum corneum permeation [12]. As seen in the transmission electron microscopy (TEM) image (Figure 1b), the Res-NLPs exhibited spherical or quasi-spherical shapes with clearly visible bilayer structures. The uniform particle size observed by TEM was consistent with the narrow size distribution (PDI < 0.15) measured by the dynamic light scattering method (DLS). The drug loading (DL) and encapsulation efficiency (EE) value of the Res-NLPs were 5.8% ± 0.2% and 90.9 ± 1.6%, respectively. The high DL and EE mean that the transdermal drug delivery system has the ability to create a high concentration gradient with the skin tissue, which could facilitate drug delivery to the skin [28,29].

The Res-NLPs exhibited excellent stability at 4 °C. After storage at 4 °C for 90 days, the Res-NLPs showed a slightly increased particle size from 55.6 nm to 66.8 nm (Figure 1c), with negligible aggregation and marginally increased PDI. In the meanwhile, the EE of the Res-NLPs almost remained unchanged during storage, staying above 80% (Figure 1d). The high zeta potential, suitable cholesterol content, and sufficiently small PDI contributed to the high stability of the bilayer membrane structure, which effectively decrease the possibility of self-aggregation during storage and thus improve the stability [30]. Overall, the above results indicate that the developed Res-NLPs had the advantages of uniform and small particle size, high DL and EE, and good stability, and therefore suitable for industrially translatable transdermal application.

### 2.2. In Vitro Release

The release profiles of Res encapsulated NLPs and free Res are shown in Figure 1e. Compared to the free Res which rapidly released 91.5% content in the first 12 h, the Res-NLPs showed a burst release in the beginning followed by a sustained release afterward. Only 44.7% and 57.0% of Res were released from the Res-NLPs after 12 h and 72 h, respectively. These results suggest that NLPs encapsulation can significantly prolong the release of Res, possibly due to the depot effect [31]. This could effectively facilitate the release of the loaded active ingredient at the target site, thus allowing the loaded drug to exert a sustained pharmacological effect over a longer period.

### 2.3. Transdermal Performance

**Figure 2:**
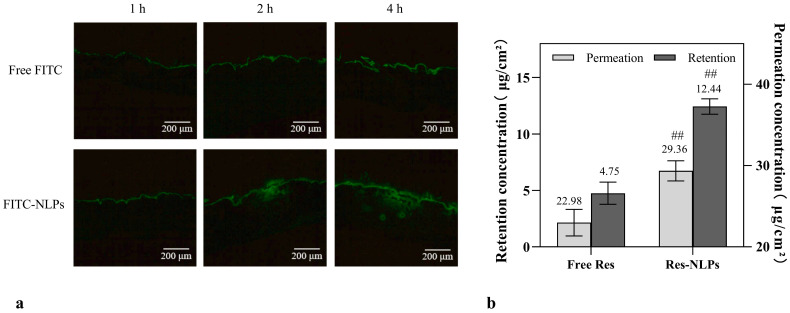
(**a**) Fluorescence microscope images showing in vivo transdermal delivery process. The porcine skin was treated with 10 μg/mL free FITC or FITC-loaded NLPs with equal FITC concentration for up to 4 h, Scale bar: 200 μm. (**b**) Cumulative percutaneous permeation and retention of Res after 24 h transdermal delivery. Mean ± SD (*n* = 3). ^##^ *p* < 0.01, compared with the free Res group.

The transdermal permeation behavior of NLPs was further investigated. Figure 2a shows that fluorescein isothiocyanate (FITC, green fluorescence) labeled NLPs penetrated deeper into the skin compared with the free FITC. As seen in Figure 2b, the cumulative Res skin retention of the Res-NLPs after 24 h was 2.62 times higher than that of the free Res (*p* < 0.01), and the responding cumulative Res permeation was 1.28 times more efficient than that of the free Res (*p* < 0.01), which means that the Res-NLPs tend to be more localized in the skin layer than the free Res. These results demonstrate that the developed Res-NLPs greatly improved the percutaneous permeability and retention of Res in the skin. The outermost stratum corneum plays a vital role in limiting Res skin permeating. NLPs could enhance transdermal permeation because NLPs have similar compositions as skin cells, and thus could permeate the skin more effectively through fusion, lipid exchange, and hydration [13,32]. In addition, the particle size of our Res-NLPs was less than 100 nm, which is a suitable size for skin surface adhesion and stratum corneum penetration [12]. Therefore, the prepared Res-NLPs could improve transdermal permeation and retention of Res, allowing for greater absorption and uptake of Res by target cells.

### 2.4. Cell Safety Evaluation

**Figure 3:**
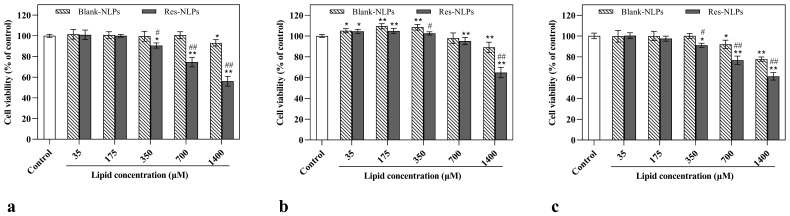
In vitro cytotoxicity of the blank NLPs, Res-NLPs against (**a**) HaCaT, (**b**) HSF and (**c**) B16F10 cells detected by CCK-8. The cells were treated with the blank NLPs or Res-NLPs at the same lipid concentrations over a wide range (35~1400 μM). The cells treated with DMEM only served as the control group. Mean ± SD (*n* = 3). * *p* < 0.05, ** *p* < 0.01, compared with the control group, ^#^ *p* < 0.05, ^##^ *p* < 0.01, compared with the blank NLPs group.

The cytotoxicity of the Res-NLPs was determined in immortalized human keratinocytes (HaCaT, typical epidermis cells), human skin fibroblasts (HSF) and murine melanoma (B16F10) cells. As shown in Figure 3, the above 80% cell viability shows that the blank NLPs and Res-NLPs displayed negligible cytotoxicity against HaCaT, HSF and B16F10 cells within the lipid concentration range of 35~175 μM. Once lipid concentration exceeded 350 μM, dose-dependent cytotoxicity was observed in the blank NLPs and Res-NLPs. The Res-NLPs were more cytotoxic than the blank NLPs within the lipid concentration range of 350~1400 μM. This result indicates high levels of Res encapsulated in high concentrations of Res-NLPs are toxic to cells, while low concentrations of Res are not [33,34]. Furthermore, above 80% cell viability was observed in all three types of cells treated with the blank NLPs at high lipid concentrations (350~700 μM), suggesting the good cell safety of the materials utilized for NLPs preparation. In addition, in Figure S1, the free Res and Res-NLPs both showed limited cytotoxicity against HaCaT, HSF and B16F10 cells within the Res concentration range of 5~50 μM. Lower toxicity (*p* < 0.05) was observed in the Res-NLPs at Res concentration between 100 and 200 μM, compared with the free Res at the same Res concentration, indicating that encapsulation of NLPs could effectively reduce the cytotoxicity of the drug. Notably, the survival rates of HSF cells treated with the blank NLPs or Res-NLPs (lipid concentration 35~350 μM) were all higher than 100%, while the free Res did not show such promoting effect. These results suggest that at a lipid concentration of 35~350 μM, the NLPs, rather than the Res, can promote the proliferation of HSF cells (Figure 3b). In addition, this stimulative effect was overshadowed by the toxicity of NLPs when the lipid concentration exceed 350 μM. We hypothesize that the phospholipids in liposomes were absorbed by HSF cells as nutrients and thus promote cell growth. This is consistent with the previous findings [22,35]. To sum up, the Res-NLPs displayed relatively high cell safety, exhibiting almost no toxicity to the selected HSF, HaCaT and B16F10 within a wide lipid concentration range (35~175 μM). This result can be used as a reference for subsequent experiments.

### 2.5. Enhanced Cellular Uptake of the Res-NLPs by Skin Cells

**Figure 4:**
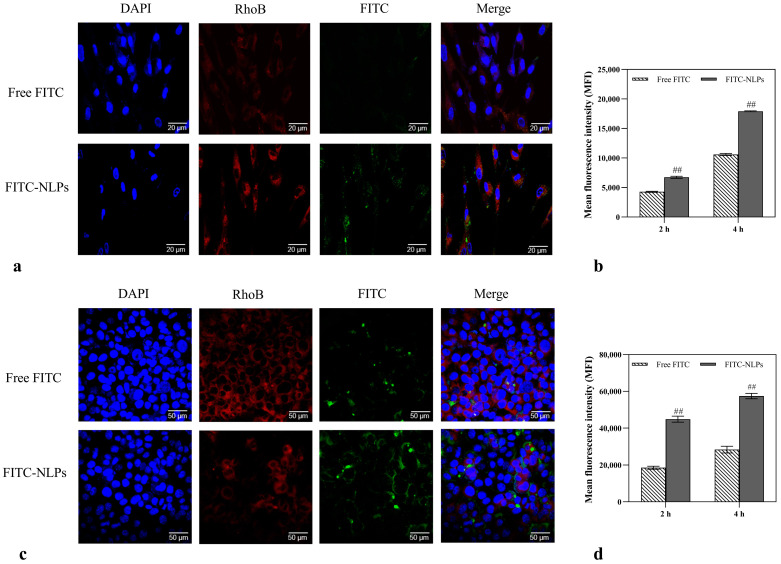
CLSM images of (**a**) HSF cells (Scale bar: 20 μm) and (**c**) B16F10 cells (Scale bar: 50 μm) showing the subcellular distribution of FITC. The cells were treated with 2 μg/mL free FITC and equal FITC concentration of FITC-NLPs for 4h. DAPI (blue) and RhoB (red) were utilized to label the nucleus and cytoplasm, respectively. Cellular uptakes of FITC by (**b**) HSF cells and (**d**) B16F10 cells were further analyzed by FCM and presented as relative mean fluorescence intensity (MFI). The FCM samples were analyzed after treating cells with free FITC or FITC-NLPs at a fixed FTIC dosage of 2 μg/mL for 2 h or 4 h. Mean ± SD (*n* = 3). ^##^ *p* < 0.01, compared with the free FITC group.

Enhancing the uptake of Res into cells could enhance its bioavailability [36]. To evaluate the uptake of NLPs by the target skin cells, the cellular uptake of the NLPs was investigated in two cell models, HSF and B16F10, which have been commonly utilized as cell models for the evaluation of anti-aging and brightening effects of skin [37,38]. The fluorescent FITC was loaded into NLPs as a trace molecule for intracellular uptake analysis of NLPs by flow cytometry (FCM) and confocal laser scanning microscope (CLSM). As shown in the CLSM images, the FITC (green) fluorescence intensity in HSF cells (Figure 4a) and B16F10 cells (Figure 4c) treated with the FITC-loaded NLPs were both considerably higher than those treated with the free FITC at an equivalent concentration. The enhanced cellular uptake via NLPs was further quantified by FCM analysis. The mean fluorescence intensity of the HSF cells (Figure 4b) and B16F10 cells (Figure 4d) treated with the FITC-NLPs for 4 h were 1.7 and 2.0 times higher than those treated with the free FITC (*p* < 0.01), respectively. The CLSM and FCM results suggest that NLPs encapsulation strongly facilitated efficient intracellular drug delivery to a variety of relevant skin cells. This is consistent with the findings of Tian et al. that NLPs could promote the uptake of the loaded component by relevant cells [18]. NLPs, as small particles within the range of nanometers, could enter cells through pinocytosis, clathrin-mediated endocytosis, and caveolae-mediated endocytosis [39]. The enhanced cellular uptake of the prepared NLPs also can be explained by the similarity of the lipid bilayer structure of NLPs to the cell structure, and the biocompatible membrane materials employed such as phospholipids and cholesterol can enhance the affinity of the Res-NLPs for both cell types [40]. In general, Res encapsulation by NLPs can greatly enhance the Res uptake by relevant cells.

### 2.6. The Res-NLPs Improved Cellular Antioxidant Activity

**Figure 5:**
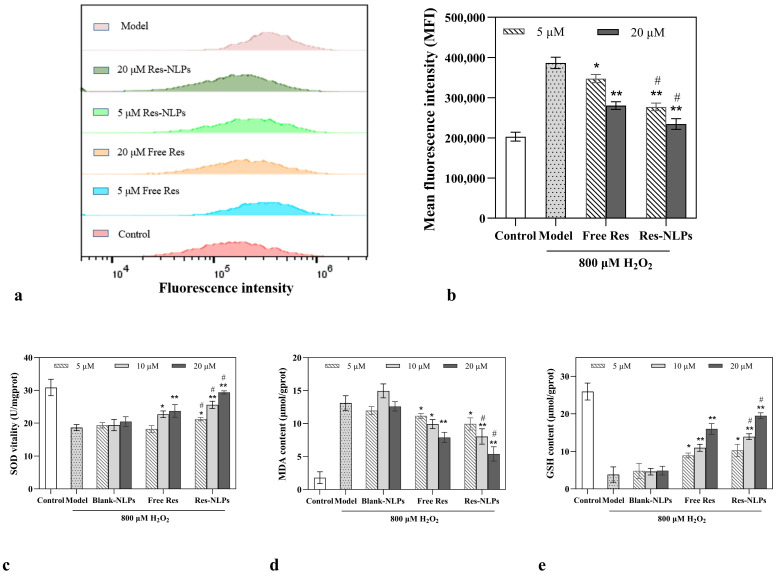
The protective effect of Res. Intracellular ROS level of HSF cells treated with the free Res or equal Res concentration of Res-NLPs for 24 h quantitatively analyzed by FCM and presented as (**a**) representative histogram plots and (**b**) relative mean fluorescence intensity (MFI). The intracellular (**c**) SOD vitality, (**d**) MDA and (**e**) GSH contents of HSF cells treated with the blank NLPs (equal lipid concentration as the Res-NLPs), free Res or equal Res concentration of Res-NLPs measured by the SOD, MDA and GSH Assay Kits. HSF cells were damaged by 800 μM H_2_O_2_ and treated with free Res or Res-NLPs at different Res concentrations (5~20 μM). Cells were treated with DMEM only as a control treatment. Mean ± SD (*n* = 3). * *p* < 0.05, ** *p* < 0.01, compared with the model group; ^#^ *p* < 0.05, compared with the free Res group.

As shown in Figure 5a,b, the free Res within a concentration range of 5~20 μM significantly reduced the intracellular reactive oxygen species (ROS) level in a concentration-dependent manner. The Res-NLPs exhibited remarkably stronger antioxidant activity than the free Res (*p* < 0.05). The antioxidant activity was further evaluated by measuring the synthesized superoxide dismutase (SOD), malondialdehyde (MDA) and glutathione (GSH). SOD and GSH are common antioxidants that can inhibit and eliminate harmful substances (such as MDA) produced by metabolism and repair damaged cells [41]. Figure 5c shows that the Res-NLPs improved SOD activity more than the free Res at the same Res concentration (*p* < 0.05). In addition, compared with the free Res of the same concentration, MDA synthesized by HSF cells treated with the 10 μM and 20 μM Res-NLPs decreased by 18.8% and 31.6% (*p* < 0.05), respectively (Figure 5d). Compared to the free Res group, GSH synthesis was increased by 26.8% and 21.9% after treating HSF cells with the Res-NLPs at Res concentrations of 10 μM and 20 μM (*p* < 0.05), respectively (Figure 5e). In sum, Res can effectively decrease ROS levels in cells, as well as regulate the synthesis of MDA, SOD, and GSH, thus achieving cellular antioxidant effects. Furthermore, encapsulating Res into NLPs remarkably can enhance the cellular antioxidant capacity of the Res. The enhanced antioxidant effect is due to the improved intracellular delivery of Res via NLPs. ROS induces and accelerates the skin aging process by activating a number of signaling pathways that lead to the inhibition of collagen production, synthesis and activation of human matrix metalloproteinases (MMPs) which are responsible for connective tissue degradation [42]. Thus, the Res-NLPs could theoretically enhance the anti-aging effect of Res.

### 2.7. Cellular Anti-Aging Study

**Figure 6:**
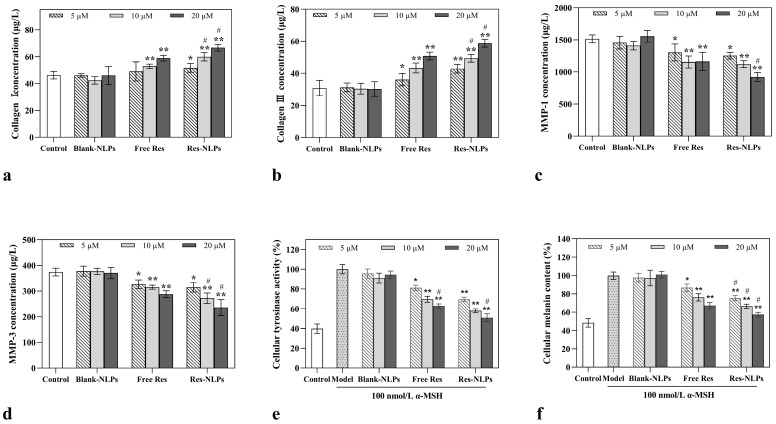
The anti-aging and skin-brightening effects of Res at the cellular level. (**a**) Collagen I, (**b**) collagen III, (**c**) MMP-1 and (**d**) MMP-3 secreted by HSF cells without treatment (served as the control group), or treated with the blank NLPs (equal lipid concentration as the Res-NLPs), the free Res or the Res-NLPs with various Res concentrations for 24 h. Inhibition of (**e**) tyrosine activity and (**f**) melanin production in B16F10 cells treated with DMEM only (served as the control group), blank NLPs (equal lipid concentration as the Res-NLPs), the free Res or the Res-NLPs with various Res concentrations for 24 h. Except for the control group, B16F10 cells in all groups were both treated with 100 nM α-melanocyte-stimulating hormone (α-MSH). B16F10 cells treated with α-MSH only served as the model group. Mean ± SD (*n* = 3). * *p* < 0.05, ** *p* < 0.01, compared with the control group (In Figure 6e, f: compared with the model group); ^#^ *p* < 0.05, compared with the free Res group.

Cellular anti-aging studies of the Res-NLPs were conducted to verify the anti-aging effectiveness of the Res-NLPs. As shown in Figure 6a,b, compared to the control group, both the free Res and Res-NLPs remarkably promoted the synthesis of collagen I and collagen III within the concentration range of 5~20 μM. Moreover, this promotion effect was enhanced with the increase in Res concentration. Compared with the free Res, the synthesis of collagen I and collagen III by HSF cells were considerably increased by 13.1% and 15.7% (*p* < 0.05) after treatment with the Res-NLPs at the same Res concentration (20 μΜ), respectively. In addition, Figure 6c,d show that compared with the control group, both the free Res and Res-NLPs notably inhibited the secretion of human matrix metalloproteinases I (MMP-1) and human matrix metalloproteinases III (MMP-3) (*p* < 0.05) by HSF cells in a concentration-dependent manner within a wide concentration range (5~20 μM). Compared with the free Res, the secretion of MMP-1 and MMP-3 by HSF cells after treatment with the Res-NLPs at Res concentrations of 20 μM for 24 h were substantially reduced by 20.7% and 18.1% (*p* < 0.05), respectively. Collagen I and collagen III are important extracellular matrix components that play an important role in skin aging by providing tensile strength and resistance to plastic deformation [43]. MMP-1 and MMP-3 are two typical members of MMPs responsible for degrading collagen and elastin, leading to skin aging and wrinkle formation [44]. The abovementioned results show that the Res-NLPs substantially enhanced the effect of Res on promoting the synthesis of collagen I and collagen III, and inhibiting the secretion of MMP-1 and MMP-3. In other words, the Res-NLPs exhibit a stronger capability to enhance collagen synthesis and inhibit collagen breakdown compared to the free Res.

### 2.8. Cellular Skin-Brightening Study

The cellular skin-brightening efficacy of the Res-NLPs was further investigated, focusing on tyrosine activity as well as melanin production. In Figure 6e,f, the melanin production and tyrosinase activity of the model group were drastically increased after melanocyte-stimulating hormone treatment, indicating that the model was successfully constructed. As shown in Figure 6e, compared with the control group, both the free Res and Res-NLPs significantly inhibited tyrosinase activity (*p* < 0.05), while the inhibitory effect of the Res-NLPs was stronger than that of the free Res. Specifically, compared with the free Res group, the tyrosine activity of B16F10 cells was prominently reduced by 16.7% and 18.9% (*p* < 0.05) after treatment with the Res-NLPs at Res concentrations of 10 and 20 μM, respectively. Furthermore, Figure 6f shows that both the free Res and Res-NLPs suppressed melanin production in B16F10 cells in a concentration-dependent manner within the concentration range tested (5~20 μM). After treatment with the Res-NLPs at Res concentrations of 10 and 20 μM, the melanin generation in B16F10 cells was reduced by 12.6% and 14.7% (*p* < 0.05) compared to the free Res group, respectively.

The color of human skin is determined by the amount, distribution, and extent of melanin in the epidermis [45]. Thus, inhibition of melanin production leads to a skin-brightening effect [37]. In addition, as reported in many studies, melanogenesis is a complex pathway in which tyrosinase is the key rate-limiting enzyme and the only necessary enzyme [46]. Res could affect the posttranscriptional regulation of melanogenic genes and inhibit mRNA expression of tyrosinase, tyrosinase-related proteins 1 and 2 [47]. The results of the above study validate that Res could be served as a tyrosine inhibitor to reduce melanin production [7]. Moreover, the Res-NLPs could enhance this brightening effect, which is due to the enhanced cellular uptake of Res by NLPs encapsulation.

### 2.9. A 3D Skin Model Efficacy Study

#### 2.9.1. Anti-Aging

To systematically investigate the dermal efficacy of the Res-NLPs, a 3D skin model was used to mimic human skin. A 3D skin model is a novel in vitro skin model with biochemical characteristics, with mechanical and structural properties almost identical to in vivo physiological state [48]. Since photoaging is the key factor for aging, the UV-stimulated 3D skin model was used in this study to simulate the aging process.

**Figure 7:**
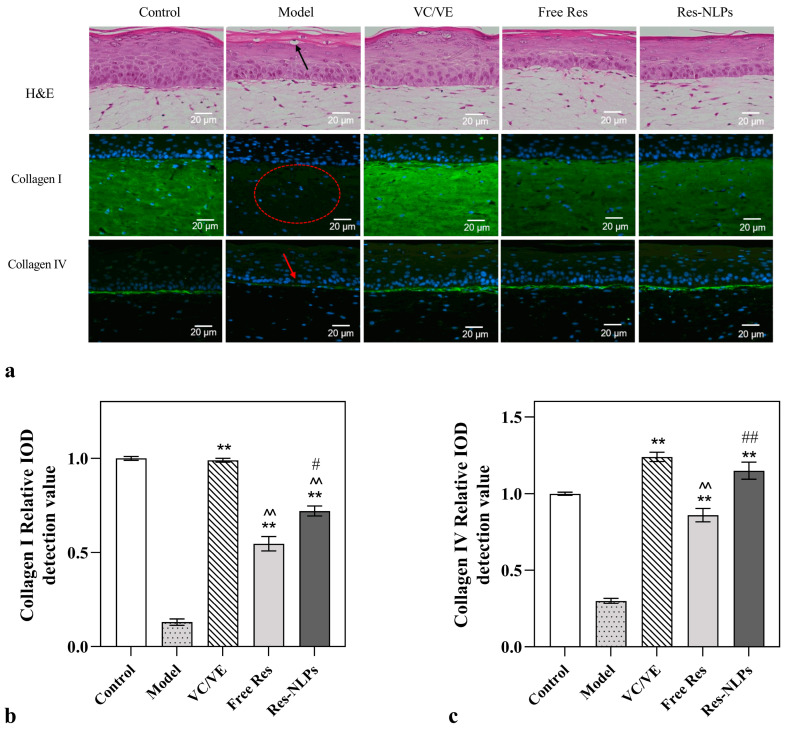
The anti-aging effect of Res studied using the 3D skin models. (**a**) The H&E staining and fluorescent immunization images of collagen I and collagen IV in the 3D skin model treated with 20 μL 100 μg/mL VC and 7 μg/mL VE, 20 μL 300 μg/mL free Res or Res-NLPs with the same Res concentration as the free Res, respectively. The relative value of synthesized (**b**) collagen I and (**c**) collagen IV were quantified by immunofluorescence microscopy. Except for the control group (without treatment), the model group, the VC/VE group, the free Res group and the Res-NLPs group were all subjected to continuous irradiation of UVA (35 J/cm^2^) and UVB (50 mJ/cm^2^). The 3D skin model treated with irradiation of UVA and UVB served as the model group. Mean ± SD (*n* = 3). ** *p* < 0.01, compared with the model group; ^^^^ *p* < 0.01, compared with the VC/VE group; ^#^ *p* < 0.05, ^##^ *p* < 0.01, compared with the free Res group.

As shown in Figure 7a, the histomorphology results show that after hematoxylin and eosin staining (H&E), the number of live epidermal cell layers was significantly reduced and the stratum corneum was loosened in the model group compared with the control group, indicating the successful construction of the skin aging model. The free Res and Res-NLPs both improved the aging damage status to different degrees, while the anti-aging effect of the Res-NLPs was stronger than that of the free Res. Furthermore, the immunofluorescence test results suggest that more collagen I and collagen IV (both shown as green fluorescence) were detected in the 3D skin after treatment with the Res-NLPs than the free Res (Figure 7a). As shown in Figure 7b,c, the synthesis of collagen I and collagen IV in the Res-NLPs group were significantly increased by 31.7% and 33.7% (*p* < 0.05), respectively, compared with the free Res group. Notably, collagen IV in the 3D skin models after treatment with the Res-NLPs was almost comparable to that in the VC/VE group (*p* > 0.05), which served as a positive control. Overall, these results prove that the Res-NLPs can effectively enhance the anti-aging effects of Res in the 3D skin models as well.

#### 2.9.2. Skin-Brightening

**Figure 8:**
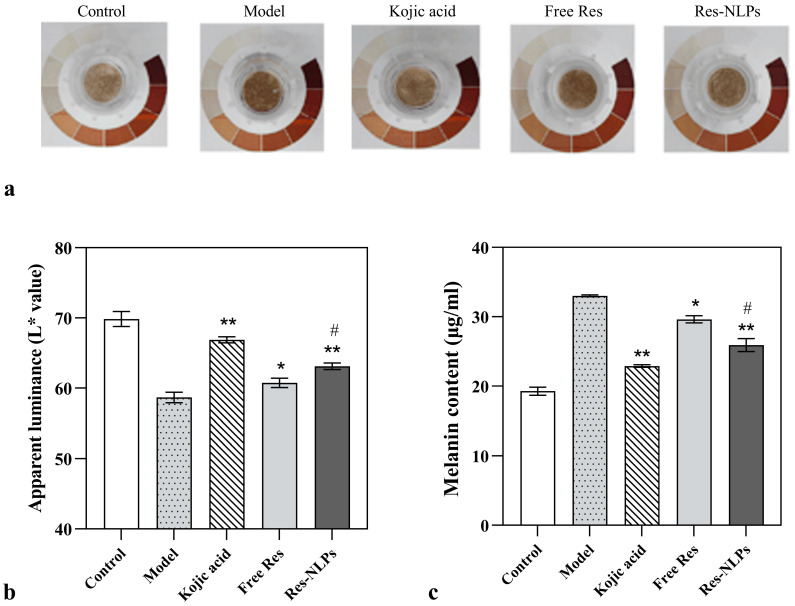
The skin-brightening effect of Res studied using the 3D melanin skin models. (**a**) apparent colorimetric, (**b**) apparent luminance and (**c**) melanin contents of the 3D skin model treated with 20 μL 500 μg/mL kojic acid, 300 μg/mL free Res or Res-NLPs with the same Res concentration as the free Res, respectively. The 3D skin model without treatment served as the control group. The model group, the kojic acid group, the free Res group and the Res-NLPs group were treated with UVB irradiation (50 mJ/cm^2^) daily, while the control group was not irradiated with UVB and only the culture solution was changed daily. The 3D skin model treated with UVB irradiation only served as the model group. Mean ± SD (*n* = 3). * *p* < 0.05, ** *p* < 0.01, compared with the model group; ^#^ *p* < 0.05, compared with the free Res group.

The successful construction of the 3D skin-brightening model was confirmed by the notably darker apparent chromaticity of the model group after UVB stimulation. Res can inhibit tyrosine activity and inhibit melanin production, thus improving the skin’s apparent luminance and brightness [45]. The apparent colorimetric increase was in the order of Res-NLPs group > free Res group > model group (Figure 8a). In addition, the apparent luminance was increased by 3.5% and 7.6% in the free Res and Res-NLPs groups (*p* < 0.05), respectively, compared with the model group (Figure 8b). There was a significant difference between the free and Res-NLPs group (*p* < 0.05). After treatment with the free Res and Res-NLPs, the melanin content test result shows that the amount of melanin synthesis was reduced by 10.3% and 21.5% compared to the model group (*p* < 0.05), respectively (Figure 8c). The effect study results of the Res-NLPs on the 3D skin model imply that the Res-NLPs could enhance the skin-brightening effect of Res on the 3D skin model. This is in agreement with our previous results on cellular-level skin-brightening studies. This not only further demonstrates the facilitative effect of NLPs on the anti-aging effects of Res, but also illustrates the feasibility of 3D skin models as an in vitro research tool to study the effects of drugs on the skin [49].

### 2.10. Human Skin Efficacy Study

Systematic studies on the anti-aging and brightening effects of Res on human skin, especially clinical trials are limited [24]. Therefore, research on the efficacy of Res on human skin is urgently necessary to provide enough guidance as well as the theoretical basis for its application in the field of human skin care. To further systematically study the anti-aging and skin-brightening effects of Res, the skin-care effects of the Res-NLPs were carried out on human skin.

**Figure 9:**
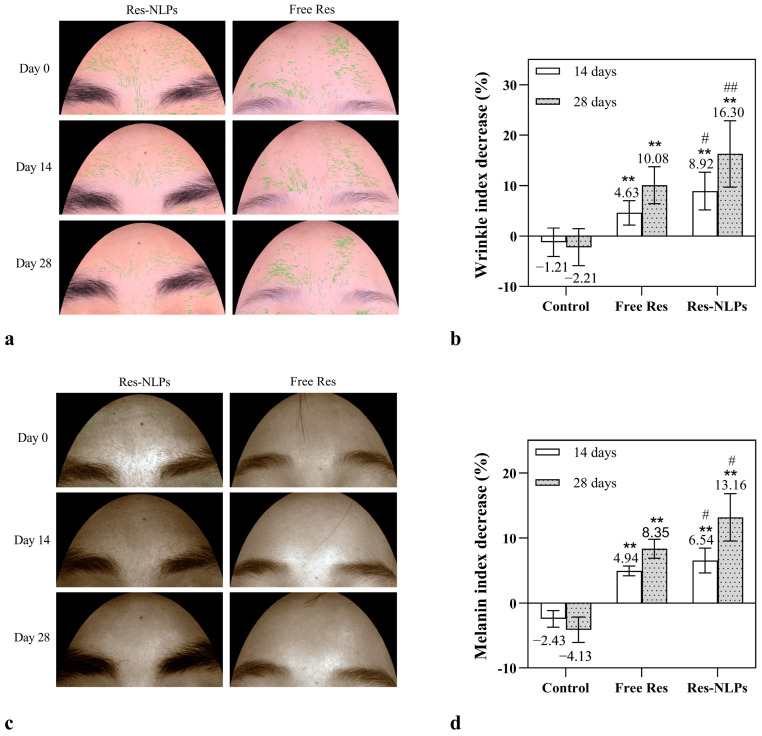
The anti-aging and skin-brightening effects of Res studied on human skin. (**a**) Representative photographs of wrinkle imaging of Chang’e skin detector on the forehead area of volunteers before and after 2 and 4 weeks of treatment with the free Res or Res-NLPs with the same Res concentration as the free Res. (**b**) The decrease (%) in wrinkle index of volunteers after 2 and 4 weeks of treatment with the Res-NLPs or free Res. (**c**) Representative pictures of melanin imaging of Chang’e skin detector in the forehead area of volunteers after 2 and 4 weeks of using the Res-NLPs or free Res. (**d**) The decrease (%) in melanin index of after 2 and 4 weeks of using the Res-NLPs or free Res. Mean ± SD (*n* = 11). ** *p* < 0.01, compared with the control group; ^#^ *p* < 0.05, ^##^ *p* < 0.01, compared with the free Res group.

As shown in Figure 9a,c, both the free Res and Res-NLPs groups of subjects experienced a reduction in forehead wrinkles and melanin deposition over time, with the Res-NLPs exhibiting a noticeably stronger anti-wrinkle and skin-brightening impact than the free Res. The anti-wrinkle and skin-brightening effects were further quantified and shown in Figure 9b and Figure 9d, respectively. The wrinkle and melanin index decrease percentages in the control group were both negative, indicating that the skin wrinkles and melanin deposition became more obvious in the control group with the treatment of the blank cream during the experiment. On the contrary, the percentages of wrinkle and melanin index decrease in the Res-NLPs group and the free Res group were positive and substantially different from the control group. It is noteworthy that the wrinkle index decrease percentages of the Res-NLPs group at 14 and 28 days were 8.9% and 16.3%, respectively, which were distinctly (*p* < 0.05) higher than that of the free Res group (4.6% and 10.1%, respectively). In addition, after 14 and 28 days, the melanin index decrease percentages of the Res-NLPs group were 6.5% and 13.2%, respectively, being significantly (*p* < 0.05) higher than that of the free Res group (4.9% and 8.4%, respectively). These results clearly demonstrate that the Res-NLPs displayed stronger anti-wrinkle and skin-brightening effects than that of the free Res. This enhancement effect is derived from the fact that the Res-NLPs could improve the transdermal permeability of Res and its ability to be taken up by relevant target cells. In addition, the sustained release nature of the Res-NLPs is also helpful for extending its pharmacological effect for a longer period.

## 3. Materials and Methods

### 3.1. Materials

Soybean lecithin was obtained from Shanghai Taiwei Pharmaceutical Co., Ltd. (Taiwei, Shanghai, China). Res, Cholesterol, 1,2-propanediol, anhydrous ethanol, methanol and glycerol were obtained from Sinopharm (Sinopharm, Beijing, China). PEG-40 hydrogenated castor oil (CO40) was purchased from BASF (BASF, Ludwigshafen, Germany). Fluorescein isothiocyanate (FITC), 4′,6-Diamidine-2′-phenylindole dihydro-chloride (DAPI), rhodamine B isothiocyanate (RhoB), α-melanocyte-stimulating hormone (α-MSH), octyl dodecanol (tritonX-100), L-dopa, vitamin C (VC), vitamin E (VE) and kojic acid were purchased from Sigma (Sigma, St. Louis, MO, USA).

Fetal bovine serum (FBS), Dulbecco’s modified eagle medium (DMEM), phosphate-buffered saline (PBS, pH 7.4), penicillin, streptomycin, trypsin-EDTA and dimethyl sulfoxide (DMSO) were purchased from Gibco (Gibco, New York, NY, USA).

### 3.2. Cell Culture

HaCaT (American Type Culture Collection, VA, USA), HSF (SynthBio, Hefei, China) and B16F10 (SynthBio, Hefei, China) were all cultured in DMEM with 10% FBS and 1% penicillin/streptomycin. The cells were incubated in a humidified atmosphere of 5% CO_2_ at 37 °C.

### 3.3. Preparation and Characterization of the Res-NLPs

The Res-NLPs were prepared using a high-pressure homogenization technique (AMH-3, Microjet high pressure homogenizer, Suzhou Antosi Nano Technology Co., Ltd., Suzhou, China). Briefly, 0.8% (*w*/*w*) Res was homogeneously distributed in 4% (*w*/*w*) 1.2-propanediol to obtain phase A. Phase B was obtained by homogenizing 0.2% (*w*/*w*) cholesterol with 4% (*w*/*w*) 1.2-propanediol, 2% (*w*/*w*) anhydrous ethanol and 8% (*w*/*w*) soybean lecithin. Phase C was prepared by dissolving 4% (*w*/*w*) PEG-40 hydrogenated castor oil and glycerol 10% (*w*/*w*) in distilled water. Subsequently, phase A was added to phase B and stirred at 45 °C for 30 min to obtain the A-B mixed phase. Phase C was added to the A-B mixed phase, stirred for 45 min at 45 °C, and then homogenized three times at 900 bar. The Res-NLPs were purified by ultrafiltration (MWCO 30 kD_a_, Amicon Ultra, Millipore, Billerica, MA, USA) at 12,000 g for 30 min to obtain concentrated Res-NLPs (repeated three times). The FITC-loaded NLPs (FITC-NLPs) or blank NLPs were prepared following the same method as described above, replacing Res with FITC or removed directly.

The zeta potential, PDI as well as the particle size of the Res-NLPs were determined using a Zetasizer/Nano-ZS90 instrument (Malvern Instruments, Malvern, UK) at 25 °C with ultra-pure water as a dispersing medium. The morphology of the Res-NLPs was observed by TEM (HT7700, Hitachi, Tokyo, Japan).

The content of Res was determined by high-performance liquid chromatography (HPLC). Ultrafiltration centrifugation was applied to remove the unencapsulated Res, and the EE and DL of the Res-NLPs were calculated using the following equations:

(1) $$\mathrm{DL}\;\left(\%\right)=\frac{W_{T}-W_{F}}{{\;W}_{L}{+W}_{T}{\;-W}_{F}}\;\times \;100,$$

(2) $$\mathrm{EE}\;\left(\%\right)=\frac{W_{T}-W_{F}}{W_{T}}\times 100,$$

where W_L_ refers to the lipid content in the Res-NLPs, W_T_ is the total Res content, and W_F_ is denoted as the unloaded Res content in the Res-NLPs.

The freshly prepared Res-NLPs were stored at 4 °C for stability studies. The particle size, PDI and EE of the Res-NLPs were measured at regular time intervals over a period of 3 months.

### 3.4. In Vitro Release Study

The in vitro release of Res was determined by dialysis. A sample of 4 mL Res-NLPs (Res concentration 4 mg/mL) and an equal volume of Res suspension with the same Res concentration were packed in dialysis tubing (MWCO 14 kDa, Biosharp, CN, USA) and immersed in 60 mL PBS (pH = 7.4) containing acetonitrile (40%, *v*/*v*) that met sink condition (120 rpm, 37 °C). One milliliter of the samples was taken at predetermined time intervals (1, 2, 4, 6, 8, 12, 24, 36, 48 and 72 h) and replenished with an equal amount of fresh release medium. The concentration of Res released was determined by HPLC.

### 3.5. In Vitro Skin Permeation

The in vitro transdermal properties of the Res-NLPs were investigated using the Franz diffusion cell method. To ensure the repeatability of the skin penetration experiment, each group contains three parallel samples. The back skin of Bama miniature pigs (5~6 kg body weight), which is commonly utilized as a in vitro skin model for drug penetration study, was purchased from Zhifu Yurong Biological Studio (Yantai, China). The hair follicles of the porcine skin were carefully protected to avoid damage, and subcutaneous adipose tissue was removed. The porcine skin was fixed between the supply pool and the receiving pool, with the stratum corneum oriented toward the supply pool. The Res-NLPs (1 mL, Res concentration 8 mg/mL) and free Res (1 mL, Res concentration 8 mg/mL) containing the same concentration of Res were added to the supply pool and applied uniformly to the porcine skin, respectively. In vitro transdermal assays were performed at 300 rpm and 37 °C. At various time intervals (0.5, 1, 2, 4, 6, 8, 10, 12 and 24 h), 0.5 mL sample was withdrawn from the receiving pool and replenished with an equal volume of fresh receiving solution at the same temperature. After the transdermal experiment, the porcine skin was removed and thoroughly washed with saline, cut into small pieces, and transferred to a centrifuge tube. To extract Res retention in the skin, the skin was ground with 1 mL of methanol and homogenized, ultrasonicated for 10 min followed by centrifugation. The supernatant was filtered and Res concentration was detected by HPLC. The cumulative permeation and retention of Res per unit area of skin were calculated using the following formulas:

(3) $$\mathrm{Skin}\;\mathrm{permeation}\;\mathrm{per}\;\mathrm{unit}\;\mathrm{area}\;=\frac{C_{n}\times V_{0}+\sum_{i=1}^{n-1}C_{i}\times V_{i}}{S},$$

where C_n_ and C_i_ are the Res concentrations measured at the nth and ith time points, respectively, V_0_ is the volume of each sampling, and S is the effective permeation area (2.27 cm^2^).

(4) $$\mathrm{Skin}\;\mathrm{retention}\;\mathrm{per}\;\mathrm{unit}\;\mathrm{area}=\frac{C\times V}{S},$$

where C is the concentration of Res extracted from the porcine skin, V is the total volume of methanol as the extract solution, and S is the effective permeation area (2.27 cm^2^).

In addition, to visualize the process of transdermal permeation of Res, 1 mL FITC-NLPs or free FITC solution (with the same FITC concentration) was added to the supply pool and applied uniformly to the porcine skin. During percutaneous permeation, the skin was removed at different time points (1, 2 and 4 h) and skin tissue sections were prepared using a cryotome (Thermo Scientific, HM525NX, Shanghai, China). The distribution of the FITC in the skin tissue sections was observed using fluorescence microscopy (IX71, Olympus, Tokyo, Japan).

### 3.6. In Vitro Cytotoxicity

Appropriate concentrations of HSF, HaCaT, and B16F10 cells were incubated in 96-well plates (Corning, New York, NY, USA) overnight, respectively, and then treated with the blank NLPs or Res-NLPs at the same lipid concentrations over a wide range (35~1400 μM). The cells treated with DMEM only served as the control group. After 24 h incubation, the cells were washed with PBS and the cell viability was determined using Cell Counting Kit 8 (CCK-8, Dojindo, Kumamoto, Japan).

### 3.7. Cellular Uptake Study

The uptake of the NLPs by HSF and B16F10 cells was visualized using confocal microscopy. HSF and B16F10 cells were treated with the free FITC solution (2 μg/mL) or FITC-NLPs with the same FITC concentration and incubated for 4 h. Subsequently, the cells were washed three times with cold PBS and fixed with 4% paraformaldehyde in PBS for 15 min. After fixation, the cells were treated with DAPI solution (2 μg/mL) to label the nucleus and RhoB (2 μg/mL) to label the cytoplasm, and imaged by CLSM (Olympus, FV3000, Japan) with the excitation wavelength of 405 nm, 488 nm and 561 nm, respectively.

The uptake of the NLPs by HSF and B16F10 cells was further quantitatively analyzed by FCM (FC500, Beckman Coulter, Fullerton, CA, USA). The cells were cultured for 24 h, then treated with the FITC-NLPs or free FITC solution with the same FITC concentration for 2 h or 4 h. After co-incubation, the culture medium was removed. The treated cells were washed with cold PBS, trypsinized, centrifuged, resuspended in 0.5 mL of cold PBS, and then subjected to FCM with the excitation wavelength of 488 nm.

### 3.8. Cellular Antioxidant Study

To measure the cellular ROS level, HSF cells were inoculated into 24-well plates (Corning, USA) at a density of 3 × 10^4^ cells per well and cultured for 24 h. Then these cells were treated with 800 μM H_2_O_2_ along with the free Res or Res-NLPs with the same Res concentration and incubated for 24 h. HSF cells treated with DMEM only served as the control group and HSF cells treated with 800 μM H_2_O_2_ in DMEM only served as the model group. After 24 h incubation, the cells were processed according to the manual of the ROS assay kit (Beyotime, Shanghai, China) and detected by FCM with the excitation wavelength of 488 nm.

To determine the synthesis of SOD, MDA and GSH, HSF cells were seeded into 6-well plates (Corning, USA) at a density of 3 × 10^5^ cells per well and cultured for 24 h. Then these cells were treated with 800 μM H_2_O_2_ along with the free Res, Res-NLPs with the same Res concentration (5 μM, 10 μM and 20 μM) or blank NLPs (with the same lipid concentration as the Res-NLPs) for 24 h. HSF cells treated with DMEM only served as the control group and HSF cells treated with 800 μM H_2_O_2_ only served as the model group. The contents of SOD, MDA, and GSH in the cells of different groups were measured with the SOD Assay Kit (Nanjing Jiancheng Biological, Nanjing, China), MDA Assay Kit (Nanjing Jiancheng Biological, Nanjing, China) and GSH Assay Kit (Nanjing Jiancheng Biological, Nanjing, China), respectively.

### 3.9. Cellular Anti-Aging Study

HSF cells were seeded at a density of 3 × 10^4^ cells per well in 24-well plates. After incubation for 24 h, these cells were treated with the free Res or Res-NLPs at a Res concentration of 5, 10 or 20 μM. In addition, a separate cell group was treated with blank NLPs at the same lipid concentration as the Res-NLPs. HSF cells treated with DMEM only served as the control group. After 24 h incubation, the collagen I and Collagen III content in the culture medium were determined using the Human Collagen I Assay Kit (Jiangsu Meimian Industrial Co., Ltd., Yancheng, China) and Human Collagen III Assay Kit (Jiangsu Meimian Industrial Co., Ltd., Yancheng, China), respectively. The content of MMP-1 and MMP-3 in the culture medium was determined using the human matrix metalloproteinases I assay kit (Jiangsu Meimian Industrial Co., Ltd., Yancheng, China) and human matrix metalloproteinases III assay kit (Jiangsu Meimian Industrial Co., Ltd., Yancheng, China), respectively.

### 3.10. Cellular Skin-Brightening Study

B16F10 cells in the logarithmic growth phase were seeded in 6-well plates at a density of 2 × 10^5^ cells per well and cultured for 24 h. Next, these cells were treated with 100 nmol/L α-MSH along with the blank NLPs (with the same lipid concentration as the Res-NLPs), free Res or Res-NLPs at a Res concentration of 5, 10 or 20 μM. B16F10 cells treated with DMEM only served as the control group, and B16F10 cells treated with 100 nM α-MSH only served as the model group. After 48 h culture, the culture medium was discarded, and triton X-100 was added to each well to a final concentration of 1% *v*/*v*%. The 6-well plates were frozen at −80 °C for 30 min, then thawed at room temperature and centrifuged. A sample of 100 µL of supernatant was withdrawn from each well and transferred to a 96-well plate. A sample of 100 µL 0.1% *w*/*v*% L-dopa solution was added to each well and the reaction was carried out at 37 °C for 2 h. The absorbance (A) of each well was measured by the microplate reader (Perkin Elmer, Waltham, MA, USA) at 495 nm wavelength. The relative tyrosine activity was calculated using the following formula:

(5) $$\mathrm{Tyrosine}\;\mathrm{activity}\;=\frac{A_{t}-A_{0}}{A_{m}-A_{0}}\times 100\%,$$

where A_t_, A_0_ and A_m_ are the absorbance of the drug administration group, blank group and model group, respectively.

The relative content of melanin in cells was determined by sodium hydroxide lysis. B16F10 cells in the logarithmic growth phase were seeded in a 12-well plate at a density of 1 × 10^5^ cells per well and cultured for 24 h, and then were treated in the same grouping and method as above. After 48 h culture, the culture medium was discarded and replaced with 500 µL 1 mol/L sodium hydroxide solution containing 10% DMSO. After incubation at 60 °C for 4 h, the plates were centrifuged, and 100 µL of supernatant was withdrawn from each well and transferred to a 96-well plate. The absorbance (A) of each well was measured by the microplate reader at 495 nm wavelength. The relative melanin production was calculated using the following formula:

(6) $$\mathrm{Melanin}\;\mathrm{production}\;=\frac{A_{t}-A_{0}}{A_{m}-A_{0}}\times 100\%,$$

where A_t_, A_0_ and A_m_ are the absorbance of the drug administration group, blank group and model group, respectively.

### 3.11. A 3D Skin Model Efficacy Study

#### 3.11.1. Anti-Aging

The 3D full-thickness skin model (FulKutis^®^, Guangdong Boxi Biotechnology Co., Guangzhou, China) was inoculated into 6-well plates. The 3D skin model was randomly divided into the control group, model group, VC/VE group (treated with 20 μL 100 μg/mL VC and 7 μg/mL VE), free Res group (treated with 20 μL 300 μg/mL free Res) and Res-NLPs group (treated with 20 μL Res-NLPs with the same Res concentration as the free Res group), with three replicates in each group. Surface administration was performed in a gentle circular manner. Except for the control group, the model group, the VC/VE group, the free Res group and the Res-NLPs group were all subjected to continuous irradiation of UVA (35 J/cm^2^) and UVB (50 mJ/cm^2^). The frequency of irradiation and drug administration was performed daily for a total of 4 times. At the end of drug administration, the models were transferred to Petri dishes (Corning, USA) and incubated in a humidified atmosphere of 5% CO_2_ at 37 °C. After 24 h incubation, the samples remaining on the surface of the model were cleaned with sterile PBS solution, and the residual liquid was gently wiped with a sterile cotton swab.

After cleaning, the model was fixed with 4% paraformaldehyde, and the tissue was embedded and sliced. Collagen I and collagen IV were detected by immunofluorescence, using a fluorescence microscope. The images were analyzed by Image-Pro Plus Image processing software. In addition, H&E of the sliced tissue was carried out for tissue morphology characterization.

#### 3.11.2. Skin-Brightening

A UVB-stimulated 3D melanin model (MelaKutis^®^, Guangdong Boxi Biotechnology Co., Guangzhou, China) was used to construct an in vitro skin damage model. The brightening efficacy was evaluated by measuring the three dimensions of apparent chromaticity, apparent luminance (L* value) and melanin content of the skin model. The 3D melanin model was inoculated in 6-well plates. The kojic acid group, free Res group and Res-NLPs group were treated with 20 μL 500 μg/mL kojic acid, 300 μg/mL free Res or Res-NLPs with the same Res concentration as the free Res, respectively. In this experiment, surface administration was performed in a gentle circular manner on the third and fifth day, respectively. The model group, the kojic acid group, the free Res group and the Res-NLPs group were treated with UVB irradiation (50 mJ/cm^2^) daily, while the control group was not irradiated with UVB and only the culture solution was changed daily.

The apparent colorimetric evaluation was performed by a camera (focal length = 5.8 mm, aperture = f/8, aperture F22, shutter speed = 1/80s, ISO = 1600). Subsequently, L* values were examined using a chromatic aperture aimed vertically at the surface of the models for inspection, and readings were repeated three times for each model. The models were placed in clean EP tubes and the melanin content was determined using the sodium hydroxide lysis method.

### 3.12. Human Skin Efficacy Study

Thirty-three adults between the ages of 30 and 55 years with significant facial wrinkles and melanin deposits were selected as volunteers and randomly equalized into three groups. All subjects have provided informed consent (Figure S2). The free Res (2%) and Res-NLPs (2%) were homogenized into creams, respectively, and a blank cream was used as a placebo. Each group was treated with one type of cream on the volunteers’ faces once in the morning and once in the evening. In this experiment, all subjects were not allowed to use any skin-care ingredients on their faces, and the facial data of all subjects were collected and recorded by the Chang’e Skin Decoder (Chang’e Innovation Biotechnology Co., Ltd., Wuhan, China) on day 0, day 14 and day 28, respectively, and processed for analysis.

### 3.13. Statistical Analysis

All results are shown as the mean and standard deviation from at least three independent experiments. Statistical analysis was performed with one-way ANOVA. *p*-values < 0.05 were considered statistically significant.

## 4. Conclusions

In this study, the Res-NLPs were developed as a transdermal delivery system to improve the transdermal permeability and enhance the anti-aging and skin-brightening effect of Res. The prepared Res-NLPs exhibited small and uniform nanoparticle size, good stability, high DL and EE, as well as improved transdermal permeability and enhanced cellular uptake. In addition, the systematic study at three levels (cellular, 3D skin models and human skin experiments) not only illustrated the anti-aging and skin-brightening efficacy and mechanism of Res to guide its application but also proved that the Res-NLPs can remarkably enhance the anti-aging and skin-brightening effect of Res by enhancing the skin permeation of Res and promoting the uptake of Res by relevant target cells. In conclusion, the developed Res-NLPs show their effectiveness in transdermal application with enhanced anti-aging and skin-brightening effects.

## Data Availability

No new data were created or analyzed in this study. Data sharing is not applicable to this article.

## Supplementary Materials

The following supporting information can be downloaded at: https://www.mdpi.com/article/10.3390/molecules28062738/s1. Figure S1: In vitro cytotoxicity of the free Res, Res-NLPs against (a) HaCaT, (b) HSF and (c) B16-F10 cells detected by CCK-8. The cells were treated with the free Res or Res-NLPs at the same Res concentrations over a wide range (5~200 μM). Mean ± SD (*n* = 3). * *p* < 0.05, ** *p* < 0.01, compared with the control group; ^#^ *p* < 0.05, compared with the free Res group. Figure S2. Informed Consent Statement. (a) Informed consent statement from all volunteers (Chinese version), (b) especially, informed consent statement from two volunteers whose facial information was included in the manuscript (English version).

## References

[1] Singh A.P., Singh R., Verma S.S., Rai V., Kaschula C.H., Maiti P., Gupta S.C. (2019). Health benefits of resveratrol: Evidence from clinical studies. Med. Res. Rev..

[2] Han G., Xia J., Gao J., Inagaki Y., Tang W., Kokudo N. (2015). Anti-tumor effects and cellular mechanisms of resveratrol. Drug Discov. Ther..

[3] Vestergaard M., Ingmer H. (2019). Antibacterial and antifungal properties of resveratrol. Int. J. Antimicrob. Agents.

[4] Jia Y., Shao J.-H., Zhang K.-W., Zou M.-L., Teng Y.-Y., Tian F., Chen M.-N., Chen W.-W., Yuan Z.-D., Wu J.-J. (2022). Emerging effects of resveratrol on wound healing: A Comprehensive Review. Molecules.

[5] Na J.-I., Shin J.-W., Choi H.-R., Kwon S.-H., Park K.-C. (2019). Resveratrol as a multifunctional topical hypopigmenting agent. Int. J. Mol. Sci..

[6] Farris P., Krutmann J., Li Y.-H., McDaniel D., Krolj Y. (2013). Resveratrol: A unique antioxidant offering a multi-mechanistic approach for treating aging skin. J. Drugs Dermatol..

[7] Ratz-Łyko A., Arct J. (2019). Resveratrol as an active ingredient for cosmetic and dermatological applications: A review. J. Cosmet. Laser Ther..

[8] Delmas D., Aires V., Limagne E., Dutartre P., Mazué F., Ghiringhelli F., Latruffe N. (2011). Transport, stability, and biological activity of resveratrol. Ann. N. Y. Acad. Sci..

[9] Van Smeden J., Bouwstra J.A., Agner T. (2016). Stratum corneum lipids: Their role for the skin barrier function in healthy subjects and atopic dermatitis patients. Skin Barrier Function.

[10] Babaie S., Del Bakhshayesh A.R., Ha J.W., Hamishehkar H., Kim K.H. (2020). Invasome: A novel nanocarrier for transdermal drug delivery. Nanomaterials.

[11] Tangau M.J., Chong Y.K., Yeong K.Y. (2022). Advances in cosmeceutical nanotechnology for hyperpigmentation treatment. J. Nanopart. Res..

[12] Yu Y.-Q., Yang X., Wu X.-F., Fan Y.-B. (2021). Enhancing permeation of drug molecules across the skin via delivery in nanocarriers: Novel strategies for effective transdermal applications. Front. Bioeng. Biotech..

[13] Zhou H., Luo D., Chen D., Tan X., Bai X., Liu Z., Yang X., Liu W. (2021). Current advances of nanocarrier technology-based active cosmetic ingredients for beauty applications. Clin. Cosmet. Investig. Dermatol..

[14] Bose T., Latawiec D., Mondal P.P., Mandal S. (2014). Overview of nano-drugs characteristics for clinical application: The journey from the entry to the exit point. J. Nanopart. Res..

[15] Fakhravar Z., Ebrahimnejad P., Daraee H., Akbarzadeh A. (2016). Nanoliposomes: Synthesis methods and applications in cosmetics. J. Cosmet. Laser Ther..

[16] Zarrabi A., Alipoor Amro Abadi M., Khorasani S., Mohammadabadi M.-R., Jamshidi A., Torkaman S., Taghavi E., Mozafari M.R., Rasti B. (2020). Nanoliposomes and tocosomes as multifunctional nanocarriers for the encapsulation of nutraceutical and dietary molecules. Molecules.

[17] Montoya-Álvarez M., Gonzalez-Perez J., Londoño M.E. (2022). Diabetic retinopathy treatments based on nanotechnology. Sci. Prepr..

[18] Tian L.-W., Luo D., Chen D., Zhou H., Zhang X.-C., Yang X.-L., Wang Y.-L., Liu W. (2022). Co-delivery of bioactive peptides by nanoliposomes for promotion of hair growth. J. Drug Deliv. Sci. Technol..

[19] Nikolova M.P., Kumar E.M., Chavali M.S. (2022). Updates on responsive drug delivery based on liposome vehicles for cancer treatment. Pharmaceutics.

[20] Choi M.J., Maibach H.I. (2005). Liposomes and niosomes as topical drug delivery systems. Skin Pharmacol. Physiol..

[21] Paolino D., Celia C., Trapasso E., Cilurzo F., Fresta M. (2012). Paclitaxel-loaded ethosomes^®^: Potential treatment of squamous cell carcinoma, a malignant transformation of actinic keratoses. Eur. J. Pharm. Biopharm..

[22] Han F., Luo D., Qu W., Chen D., Hong Y., Sheng J., Yang X., Liu W. (2020). Nanoliposomes codelivering bioactive peptides produce enhanced anti-aging effect in human skin. J. Drug Deliv. Sci. Technol..

[23] Wei T., Chen D., Mei H., Zhou Z., Sheng J., Liu W. (2020). Cationic Nanoliposomes Efficiently delivering phenylethyl resorcinol produce enhanced skin lightening effect. Nano LIFE.

[24] Lin M.-H., Hung C.-F., Sung H.-C., Yang S.-C., Yu H.-P., Fang J.-Y. (2021). The bioactivities of resveratrol and its naturally occurring derivatives on skin. J. Food Drug Anal..

[25] Satooka H., Kubo I. (2012). Resveratrol as a kcat type inhibitor for tyrosinase: Potentiated melanogenesis inhibitor. Bioorg. Med. Chem..

[26] Mathes S.H., Ruffner H., Graf-Hausner U. (2014). The use of skin models in drug development. Adv. Drug Deliv. Rev..

[27] Eberlin S., Silva M.S.d., Facchini G., Silva G.H.d., Pinheiro A.L.T.A., Eberlin S., Pinheiro A.d.S. (2020). The ex vivo skin model as an alternative tool for the efficacy and safety evaluation of topical products. Altern. Lab. Anim..

[28] Lane M.E., Hadgraft J., Oliveira G., Vieira R., Mohammed D., Hirata K. (2012). Rational formulation design. Int. J. Cosmet. Sci..

[29] Yang Q., Liu S., Gu Y., Tang X., Wang T., Wu J., Liu J. (2019). Development of sulconazole-loaded nanoemulsions for enhancement of transdermal permeation and antifungal activity. Int. J. Nanomed..

[30] Bondu C., Yen F.T. (2022). Nanoliposomes, from food industry to nutraceuticals: Interests and uses. Innov. Food Sci. Emerg. Technol..

[31] Xu J., Jiang S., Liu L., Zhao Y., Zeng M. (2021). Encapsulation of oyster protein hydrolysates in nanoliposomes: Vesicle characteristics, storage stability, in vitro release, and gastrointestinal digestion. J. Food Sci..

[32] Xia H., Tang Y., Huang R., Liang J., Ma S., Chen D., Feng Y., Lei Y., Zhang Q., Yang Y. (2022). Nanoliposome use to improve the stability of phenylethyl resorcinol and serve as a skin penetration enhancer for skin whitening. Coatings.

[33] Chen S., Tamaki N., Kudo Y., Tsunematsu T., Miki K., Ishimaru N., Ito H.-O. (2021). Protective effects of resveratrol against 5-fluorouracil-induced oxidative stress and inflammatory responses in human keratinocytes. J. Clin. Biochem. Nutr..

[34] Zuccari G., Alfei S., Zorzoli A., Marimpietri D., Turrini F., Baldassari S., Marchitto L., Caviglioli G. (2021). Increased water-solubility and maintained antioxidant power of resveratrol by its encapsulation in vitamin E TPGS micelles: A potential nutritional supplement for chronic liver disease. Pharmaceutics.

[35] Li J., Nan J., Wu H., Park H.J., Zhao Q., Yang L. (2022). Middle purity soy lecithin is appropriate for food grade nanoliposome: Preparation, characterization, antioxidant and anti-inflammatory ability. Food Chem..

[36] Yang C., Wang Y., Xie Y., Liu G., Lu Y., Wu W., Chen L. (2019). Oat protein-shellac nanoparticles as a delivery vehicle for resveratrol to improve bioavailability in vitro and in vivo. Nanomedicine.

[37] Shim J.H. (2021). Whitening effects of anthricin on B16F10 cells. Korean J. Pharmacogn..

[38] Wu Y., Cao K., Zhang W., Zhang G., Zhou M. (2021). Protective and anti-Aging effects of 5 cosmeceutical peptide mixtures on hydrogen peroxide-induced premature senescence in human skin fibroblasts. Skin Pharmacol. Physiol..

[39] Foroozandeh P., Aziz A.A. (2018). Insight into cellular uptake and intracellular trafficking of nanoparticles. Nanoscale Res. Lett..

[40] Kim B.-S., Na Y.-G., Choi J.-H., Kim I., Lee E., Kim S.-Y., Lee J.-Y., Cho C.-W. (2017). The improvement of skin whitening of phenylethyl resorcinol by nanostructured lipid carriers. Nanomaterials.

[41] Xiao Z., Yu X., Zhang S., Liang A. (2022). The expression levels and significance of GSH, MDA, SOD, and 8-OHdG in osteochondral defects of rabbit knee joints. BioMed Res. Int..

[42] Wang Y., Wang L., Wen X., Hao D., Zhang N., He G., Jiang X. (2019). NF-κB signaling in skin aging. Mech. Ageing Dev..

[43] Cole M.A., Quan T., Voorhees J.J., Fisher G.J. (2018). Extracellular matrix regulation of fibroblast function: Redefining our perspective on skin aging. J. Cell Commun. Signal..

[44] Park S.-Y., Byun E.J., Lee J.D., Kim S., Kim H.S. (2018). Air pollution, autophagy, and skin aging: Impact of particulate matter (PM10) on human dermal fibroblasts. Int. J. Mol. Sci..

[45] LaBerge G.S., Duvall E., Grasmick Z., Haedicke K., Galan A., Leverett J., Baswan S., Yim S., Pawelek J. (2020). Focus: Skin: Recent advances in studies of skin color and skin cancer. Yale J. Biol. Med..

[46] Pillaiyar T., Manickam M., Namasivayam V. (2017). Skin whitening agents: Medicinal chemistry perspective of tyrosinase inhibitors. J. Enzym. Inhib. Med. Chem..

[47] Kanlayavattanakul M., Lourith N. (2018). Skin hyperpigmentation treatment using herbs: A review of clinical evidences. J. Cosmet. Laser Ther..

[48] Randall M.J., Jüngel A., Rimann M., Wuertz-Kozak K. (2018). Advances in the biofabrication of 3D skin in vitro: Healthy and pathological models. Front Bioeng. Biotech..

[49] Niehues H., Bouwstra J.A., El Ghalbzouri A., Brandner J.M., Zeeuwen P.L.J.M., van den Bogaard E.H. (2018). 3D skin models for 3R research: The potential of 3D reconstructed skin models to study skin barrier function. Exp. Dermatol..

