# Supplementary material for: Systematic Study of Resveratrol Nanoliposomes Transdermal Delivery System for Enhancing Anti-Aging and Skin-Brightening Efficacy
Source: Molecules. 2023 Mar 17;28(6):2738. doi: 10.3390/molecules28062738 (PMC10058087; doi:10.3390/molecules28062738)
Supplement: Supplementary file 1 [file molecules-28-02738-s001.zip › Informed consent (English version).pdf]

Patient Consent Form for Articles Containing Patient Details and/or Images

This form provides consent for MDPI to publish details and/or images from patients. It must be completed prior to publication.

Patient details

Patient name: ___________________________________

Volunteer	History of allergy	Cutaneous history	Melanin (Day 0)	Melanin (Day 14)	Melanin (Day 28)	Wrinkle (Day 0)	Wrinkle (Day 14)	Wrinkle (Day 28)
A	without	without	17.826	17.030	16.328	1.529	1.372	1.187
B	without	without	16.374	15.758	15.384	1.307	1.239	1.139

If a representative (e.g. parent, guardian, or next of kin) is signing on the patient's behalf in the event of the patient being underage, deceased or deemed unfit to give legal consent:

Name of patient representative: ___________________________________

Relationship of representative to patient: ___________________________________

By signing this form, I confirm that I have the authority to represent the patient and provide authorization on their behalf.

Article details

Article title: Systematic study of resveratrol nanoliposomes transdermal delivery system for enhancing anti-aging and skin-brightening efficacy

Journal: Molecules

Authors: Wei Liu, Xinchao Zhang, Siyuan Chen, Dan Luo, Dan Chen, Hong Zhou, Shuting Zhang, Xuan Chen

Declaration by patient or their representative

I, the patient named above or the patient's representative, have read the abovenamed article in full (including text, figures, and supplementary material) and agree to its publication. I am fully aware of the implications of publication and accept any associated risk. In particular, I understand that, despite anonymization, it is possible that I (or the patient) may be identified based on the details or

images contained in the article. While the authors and the publisher will make efforts to minimize this risk, confidentiality cannot be guaranteed.

I understand that the paper will be published online in open access format (using a creative commons CC BY 4.0 license, http://creativecommons.org/licenses/by/4.0), meaning that it can be downloaded, copied and reused without limitation. This include any figures, tables, and supplementary data. The primary audience for the published paper will be healthcare professionals, research academics and students from across the globe.

The final published version may differ from the one submitted to the journal due to minor revisions, changes to style, and reformatting. Publication in the journal mentioned above is not guaranteed and will take place at the discretion of the publisher, and with permission of the Editor-in-Chief (or a qualified Editorial Board member) after a peer review process.

Signing this form does not remove any of my/the patient's statutory rights to privacy. I understand that I may revoke consent at any point prior to publication, but after publication my consent can no longer be withdrawn.

I understand that I/the patient will receive no financial benefit or compensation from publication of the article.

Patient and/or representative signature(s):

____

Place, date:

_____________2023.02.01_____________________
