# Supplementary material for: Systematic Study of Resveratrol Nanoliposomes Transdermal Delivery System for Enhancing Anti-Aging and Skin-Brightening Efficacy
Source: Molecules. 2023 Mar 17;28(6):2738. doi: 10.3390/molecules28062738 (PMC10058087; doi:10.3390/molecules28062738)
Supplement: Supplementary file 1 [file molecules-28-02738-s001.zip › Informed consent (Original Chinese version).pdf]

知情同意书及承诺书

1、使用的产品为白藜芦醇纳米脂质体，早晚各一次，均匀涂抹在面部。

2、使用时间为 28 天，必须每天坚持使用。

3、志愿者需要在第 0 天、第 14 天以及第 28 天配合检测面部数据。

4、志愿者的面部数据不会用于任何商业用途，只会用于科研用途。

5、志愿者同意将这些数据用于科研用途（例如文章发表、专利撰写等）。

6、使用该产品不会有不良副作用，如出现不适，请及时停止使用。

7、如因不可抗力因素停止使用了产品，请及时联系报备。

8、我们承诺已被完全告知所有相关细节。

志愿者签名以及详细信息记录表：

编号	姓名	签名	年龄	有无皮肤病史	有无过敏	刺激测试	0	14	28
1	吴孟丽	吴孟丽		无	无	✓	✓	✓	✓
2	罗丹	罗丹		无	无	✓	✓	✓	✓
3	陈丹	陈丹		无	无	✓	✓	✓	✓
4	周洪	周洪		无	无	✓	✓	✓	✓
5	赵小静	赵小静		无	无	✓	✓	✓	✓
6	崔梅	崔梅		无	无	✓	✓	✓	✓
7	郑耀武	郑耀武		无	无	✓	✓	✓	✓
8	韩娇艳	韩娇艳		无	无	✓	✓	✓	✓
9	胡婷	胡婷		无	无	✓	✓	✓	✓
10	李娇娇	李娇娇		无	无	✓	✓	✓	✓
11	皮冰冰	皮冰冰		无	无	✓	✓	✓	✓
12	童琳	童琳		无	无	✓	✓	✓	✓
13	吴光俊	吴光俊		无	无	✓	✓	✓	✓
14	吴雨桐	吴雨桐		无	无	✓	✓	✓	✓
15	熊瑜	熊瑜		无	无	✓	✓	✓	✓

16	许恕平	许恕平	无	无	√	√	√	√
17	徐世燕	徐世燕	无	无	√	√	√	√
18	王小康	王小康	无	无	√	√	√	√
19	詹锦非	詹锦非	无	无	√	√	√	√
20	王雨涵	王雨涵	无	无	√	√	√	√
21	叶志芳	叶志芳	无	无	√	√	√	√
22	张利娟	张利娟	无	无	√	√	√	√
23	李承恩	李承恩	无	无	√	√	√	√
24	肖咏	肖咏	无	无	√	√	√	√
25	刘小兵	刘小兵	无	无	√	√	√	√
26	马雯婷	马雯婷	无	无	√	√	√	√
27	孙安媛	孙安媛	无	无	√	√	√	√
28	孔建军	孔建军	无	无	√	√	√	√
29	郑方佟	郑方佟	无	无	√	√	√	√
30	高珂	高珂	无	无	√	√	√	√
31	王建伟	王建伟	无	无	√	√	√	√
32	方俊	方俊	无	无	√	√	√	√
33	任淑媛	任淑媛	无	无	√	√	√	√

我们承诺已被告知所有细节，并且同意这些数据用于科学研究（文章、发表等）。

时间：2022 年 4 月 27 [illegible]
