# Supplementary material for: Systematic Study of Resveratrol Nanoliposomes Transdermal Delivery System for Enhancing Anti-Aging and Skin-Brightening Efficacy
Source: Molecules. 2023 Mar 17;28(6):2738. doi: 10.3390/molecules28062738 (PMC10058087; doi:10.3390/molecules28062738)
Supplement: Supplementary file 1 [file molecules-28-02738-s001.zip › Supporting Information.pdf]

Supporting Information

Systematic Study of Resveratrol Nanoliposomes Transdermal Delivery System for Enhancing Anti-Aging and Skin-Brightening Efficacy

Xinchao Zhang [1], Siyuan Chen [2], Dan Luo [3], Dan Chen [3], Hong Zhou [1], Shuting Zhang [1], Xuan Chen [1], Wangwang Lu [4,*] and Wei Liu [1,3,*]

[1] College of Life Science and Technology, Huazhong University of Science and Technology, Wuhan 430074, China
[2] College of Materials Science and Engineering, Suqian Advanced Materials Industry Technology Innovation Center, Nanjing Tech University, Nanjing 211816, China
[3] National Engineering Research Center for Nanomedicine, Huazhong University of Science and Technology, Wuhan 430075, China
[4] Guangzhou Jiyan Cosmetics Technology Co., Ltd., Guangzhou 510275, China
* Correspondence: leo.lu@yatsenglobal.com (W.L.); wliu@hust.edu.cn (W.L.)

Figure S1

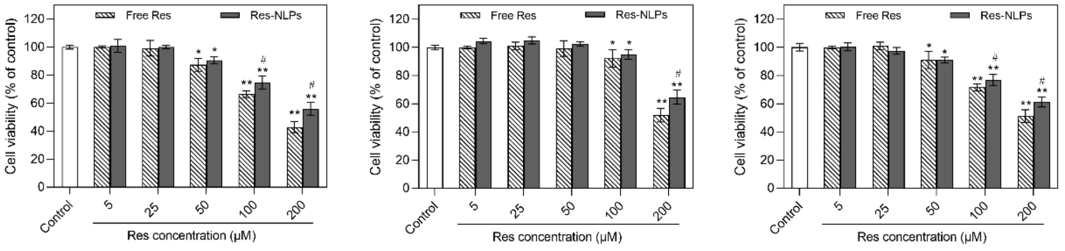

a **b** **c**

Figure S1. In vitro cytotoxicity of the free Res, Res-NLPs against (**a**) HaCaT, (**b**) HSF and (**c**) B16-F10 cells detected by CCK-8. The cells were treated with the free Res or Res-NLPs at the same Res concentrations over a wide range (5~200 μM). Mean ± SD (n = 3). $^{*}p < 0.05$, $^{**}p < 0.01$, compared with the control group; $^{\#}p < 0.05$, compared with the free Res group.

Figure S2

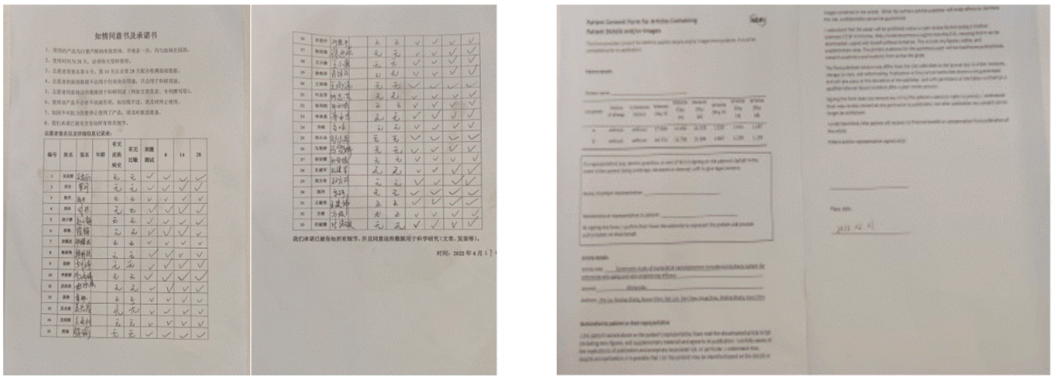

a **b**

Figure S2. Informed Consent Statement. (**a**) Informed consent statement from all volunteers (Chinese version), (**b**) especially, informed consent statement from two volunteers whose facial information was included in the manuscript (English version).
